# Parental Support and Problematic Smartphone Use: A Serial Mediating Model of Self-Esteem and Fear of Missing Out

**DOI:** 10.3390/ijerph19137657

**Published:** 2022-06-23

**Authors:** Ji-Hye Kim

**Affiliations:** College of Nursing, Woosuk University, 443 Samnye-ro, Samnye-eup, Wanju-gun 55338, Jeollabuk-do, Korea; jihye2020@woosuk.ac.kr; Tel.: +82-63-290-1749

**Keywords:** adolescents, smartphone, parental support, self-esteem, fear of missing out, mediating model

## Abstract

Based on problem behavior theory and interpersonal acceptance–rejection theory, this study aimed to examine the mediating roles of self-esteem and fear of missing out (FoMO) on the influence of parental support on adolescents’ problematic smartphone use. This study is a cross-sectional and descriptive study. A total of 260 Korean adolescents from two public middle schools were selected through convenience sampling (female, 50.4%; mean age, 13.16 ± 0.84; range age, 12~15). Participants completed self-report questionnaires assessing sociodemographic characteristics, parental support, self-esteem, FoMO, and problematic smartphone use. The collected data were analyzed using descriptive statistics, *t*-test, ANOVA, Pearson’s correlation coefficients, and mediation analysis. The findings show that self-esteem and FoMO play a serial mediating role in the relationship between parental support and adolescents’ problematic smartphone use. Specifically, parental support had a negative effect on adolescents’ problematic smartphone use by increasing self-esteem but decreasing FoMO. These results provide further guidance in the prevention of and intervention of adolescent problematic smartphone use.

## 1. Introduction

The adolescent period is a highly critical period, as it is when physical and cognitive development takes place [1]. As adolescents have developmental characteristics in which they place a high significance on building and maintaining strong bonds among peers, as well as emotional instability, weak self-control, and a strong desire to express themselves, they are more likely to rely on smartphones compared with other age groups [1,2]. According to a recent survey, Korean adolescents categorized in the problematic smartphone use (PSU) risk group accounted for 35.8%, which was the biggest number among all age groups; middle school students, in particular, scored the highest, in which 39.6% were classified in the risk group [3]. Excess use of smartphones results in negative consequences in multiple aspects such as emotional health problems, physical health problems, professional performance, social performance, and dangerous use [4,5,6]. Therefore, there needs to be social interest and research to identify potential predictors of PSU that could be leveraged in later interventions for their physical, emotional, and social development.

With increased interest in adolescent PSU, research into the early stage has mainly focused on the relationship between individuals’ psychological characteristics (personality, depression, anxiety, or stress) and PSU [5,7,8]. However, as a number of studies have recently reported that parental factors also influence not only adolescents’ personality and psychological characteristics but also their mental health and problematic behaviors, their importance has come under the spotlight [9,10,11]. According to the problem behavior theory (PBT) [12], adolescents’ problematic behaviors can be interpreted from a sociopsychological perspective; the personality system (personal values, expectations, and beliefs) and the perceived environmental system (family support, control, and model) each or interact together and influence the behavior system (drug use, drinking, general problem behavior). From the PBT point of view, adolescents’ PSU is influenced by individuals’ emotional and cognitive factors as well as environmental factors (parents, friends, etc.), which has been proven through a number of studies [7,8,9,10,11]. However, studies on the influential mechanism among the systems have been limited; hence, this study was designed to contribute to the expansion of knowledge about adolescents’ PSU through an in-depth study on the topic. Therefore, this study aimed to identify the influential mechanism among each factor while placing the focus on self-esteem and fear of missing out (FoMO) from the personality system and parental support from the environmental system. The purpose of this study was to verify the serial mediating effect of self-esteem and FoMO in the relationship between parental support and the PSU of middle school students in Korea.

### 1.1. Parental Support and PSU

According to the interpersonal acceptance–rejection theory (IPAR theory), which is formally known as the PAR theory, interpersonal acceptance–rejection is in association with individuals’ psychological adaptation [13]. For children and adolescents, in particular, parental acceptance and rejection have been identified to have a high correlation with their internalizing problems, externalizing problems, and drug and alcohol abuse [13]. Parental acceptance toward children is explained as warmth, affection, care, comfort, concern, nurturance, support, or simply love, and they are expressed through physical, verbal, and symbolic behaviors [13]. Previous studies about the relationship between parental factors and PSU support the IPAR theory [9,11,14]. Parents’ support and acceptance fill adolescents’ desire for love, affection, and a sense of belonging while helping them build a positive self-concept, posing a positive impact on their growth [15]. Moreover, an affectionate bond with parents provides social control, which plays a significant role in preventing adolescents’ problematic behaviors such as smartphone, Internet, and drug addiction [16,17,18]. Meanwhile, adolescents who perceive parental rejection show a compensatory response in which they find support online to seek psychological stability and ease negative feelings and anxiety caused by such a deficit [14,19]. Such a process can heighten the risk of PSU. It is hypothesized that parental support would be negatively related to adolescents’ PSU (hypothesis 1).

### 1.2. The Mediating Role of Self-Esteem

According to the IPAR theory, parental acceptance poses a positive influence on adolescents’ self-esteem, emotional stability, and viewpoint [20]. Parental acceptance and support help adolescents realize that they are valuable beings themselves, positively evaluating themselves [21,22]. They also meet adolescents’ social desires, influencing their establishment of a positive self-identity [21]. On the other hand, adolescents who perceive rejection from their parents have their process of self-recognition damaged, which makes them have a negative evaluation of themselves [22]. Previous studies have found that the bond between parents and their adolescent stimulates individuals’ positive psychological factors such as self-esteem, psychological stability, and emotional control, which indirectly influences addictive behaviors, such as Internet addiction or PSU [9,11,23]. Here, self-esteem is a positive or negative attitude toward one’s ego [24], and it has been emphasized as an important determinant that affects adolescents’ problematic behaviors [11,25,26,27]. Adolescents with low self-esteem tend to be immersed in their smartphones as a means to recover their self-esteem damaged from relationships offline, showing an addictive tendency [26,27]. Therefore, it is hypothesized that self-esteem would play a mediating role in the relationship between parental support and adolescents’ PSU (hypothesis 2).

### 1.3. The Mediating Role of FoMO

If adolescents perceive rejection or low support from their parents, they experience negative emotions, such as fear, anxiety, and depression [13,28,29]. FoMO refers to one’s desire to keep track of what others are doing due to the fear that one will fail to follow a trend in their group or be left out [30]. FoMO occurs when an individual lacks basic psychological needs of autonomy, proficiency, and relationship; deficiency in relationships particularly makes one fear being omitted from social relationships [30,31]. In other words, if individuals fail to satisfy basic psychological needs from their relationship with parents, they may experience heightened FoMO [28,29]. Such an unstable affection with parents expands into attachment to peer groups, which causes fear that they may miss an experience with their peers [32,33]. As adolescents have a developmental characteristic in which they prioritize the formation and maintenance of bonds within their peer groups, FoMO could be a particularly important factor. For adolescents, using social media on their smartphones is a means to express themselves freely and a process of establishing their social identity [34]. They frequently check social media for fear that they may be omitted from their social relationship with their peers, which leads to the overuse of smartphones [35,36,37]. Therefore, it is hypothesized that FoMO would play a mediating role between parental support and adolescents’ PSU (hypothesis 3).

### 1.4. The Serial Mediating Role of Self-Esteem and FoMO

Self-esteem is highly correlated with psychological characteristics such as social anxiety, isolation, and loneliness [38,39]. One with low self-esteem perceives oneself as a helpless, negative being, feeling greater social anxiety and isolation [38,39]. A longitudinal study conducted recently on the relationship between self-esteem and FoMO found that low self-esteem is highly related to FoMO [40]. In addition, previous studies have found that self-esteem is in a negative relationship with FoMO [40,41]. Therefore, it is hypothesized that self-esteem would be negatively related to FoMO and self-esteem, and FoMO would have a serial mediating mechanism in the relationship between parental support and adolescents’ PSU (hypothesis 4).

## 2. Materials and Methods

### 2.1. Study Design

This study was a cross-sectional descriptive survey designed to verify the serial mediating effect of self-esteem and FoMO in the relationship between parental support and the PSU of middle school students in Korea.

### 2.2. Subjects and Data Collection

This study was based on the original data [42]. Convenience sampling was conducted on the original data on middle school students enrolled in two middle schools located in Seoul and Gwangmyeong. Data were collected from 260 students.

Data for the study were collected from 26 November to 10 December 2018. The researcher visited the classroom where the participants were gathered and explained the purpose and method of the study, guaranteed anonymity and confidentiality, and clarified that they could withdraw their consent to participate and there would be no disadvantage for not participating in the study. The manual, guardian/participant consent form, and questionnaire were placed in an adhesive envelope and distributed to the subjects who agreed to participate in the study. Then, if the guardian and participant gave written consent to participate in the study, the participant completed the questionnaire, put it in an envelope, and sealed it. The completed questionnaire was collected by the researcher directly from each of the participants over 2 days after the questionnaire was distributed.

This study was exempted from ethical approval (No. WS-2021-21). The collection of the original data was carried out after receiving research approval from the Institutional Review Board of H University (No. HYI-18-140-1).

### 2.3. Measurements

#### 2.3.1. Sociodemographic Characteristics

Gender, age, family structure, parents’ working status, perceived economic status, and perceived health status were surveyed. For the family structure, the question “Who are you currently living with?” was used and was reclassified into “Living with both parents” and “Living with one parent/Not living with parents”. For the parents’ working status, the subjects were told to answer the question “Are your parents working?” which was reclassified into “Both of them are working” and “One of them is working/None of them are working”. For the perceived economic status, the subjects were guided to select from “High”, “Average”, and “Low”. For the perceived health status, they answered the question, “Compared to your peers, how do you feel about your health?”

#### 2.3.2. Parental Support

The Student Social Support Scale (SSSS), developed by Nolten [43] and adapted by Kim [44], was used to examine the level of recognition of parental support. Consisting of 15 items, this scale measures the level of adolescents’ recognition of emotional, informational, evaluative, and material support from their parents (e.g., “My parents patting or hugging me,” “My parents praise me for what I do well,” etc.). Each item is evaluated on a 5-point Likert scale ranging from 1 (Not at all) to 5 (Strongly agree), and a higher summed score means greater recognition of parental support. At the time of development, Cronbach’s α was 0.97, and the test–retest reliability was 0.75 [44]. In this study, Cronbach’s α was 0.955.

#### 2.3.3. Self-Esteem

The Korean Self-Esteem Scale was used [25,45]. The scale consists of a total of 10 items, 5 positive and 5 negative. Each item is evaluated on a 5-point Likert scale ranging from 1 (Not at all) to 5 (Strongly agree), and a higher summed score means greater self-esteem. At the time of development, Cronbach’s α was 0.93 [25], and it was 0.852 in this study.

#### 2.3.4. FoMO

FoMO was measured using the one-item scale developed by Riordan et al. [46]. The subjects were instructed to answer the question “Do you experience the fear of missing out by others (friends, family, etc.)” on a 5-point Likert scale ranging from 1 (Not at all) to 5 (Strongly agree); a higher score means greater fear.

#### 2.3.5. Problematic Smartphone Use

The Smartphone Addiction Proneness Scale for Youth (SAPS) was used [47]. SAPS takes difficulty in daily living, virtual life orientation, withdrawal, and tolerance as sub-domains and consists of 15 items with replies on a 4-point Likert scale (e.g., “I panic when I am unable to use my smartphone,” “School grades declined due to excessive use of smartphones”). Items 8, 10, and 13 were summed up by inverse transformation into inverse items. A higher summed score means greater problematic smartphone use. Cronbach’s α was 0.88 in the developmental study [47], while Cronbach’s α was 0.903 in the study.

### 2.4. Data Analysis

SPSS 22.0 (IBM, Armonk, NY, USA) and PROCESS macro version 4.0 (Heyes, AB, Canada) were used for analysis. Descriptive statistical analysis was performed on the demographic characteristics and major variables of the study subjects. To determine whether there is a difference in PSU according to demographic characteristics, *t*-test, ANOVA, and Scheffe test were performed. In addition, Pearson correlation analysis was performed to determine the correlation between the major variables. Then, the mediation analysis was computed by Heyes’ PROCESS macro program (model 6) to assess whether self-esteem and FoMO have serial mediating effects on the relationship between parental support and middle school students’ PSU. The significance of mediation paths was confirmed by the bootstrapping technique. To investigate the significance of the mediation path, a 95% confidence interval was applied, and the sample was extracted 5000 times for analysis.

## 3. Results

### 3.1. Differences in PSU by Sociodemographic Characteristics

The sociodemographic characteristics are shown in Table 1. Among 260 subjects, female students accounted for 50.4%. The average age was 13.16 ± 0.84. Among the subjects, 83.8% were in a family with two parents, and 60.4% of them had both parents working. In addition, 68.8% replied that their economic status was “average,” and 53.5% replied that they felt “healthy”.

Among the sociodemographic characteristics, the factor that showed a significant difference in PSU was perceived health status (F = 5.128, *p* = 0.002). It was found that those who replied “Healthy” showed a significantly higher PSU than those who replied “Very healthy” (Table 1).

### 3.2. Correlation among Main Variables

The results of analyzing the correlation between independent and dependent variables are shown in Table 2. It was found that PSU had a significant negative correlation with parental support (r = −0.269, *p* < 0.001) and self-esteem (r = −0.413, *p* < 0.001), while it had a significant positive correlation with FoMO (r = 0.404, *p* < 0.001).

### 3.3. Verification of Mediating Effects

To identify whether self-esteem and FoMO have serial mediating effects in the relationship between parental support and adolescents’ PSU, we ran a PROCESS macro analysis (model 6). Perceived health status, which showed a significant difference in PSU among sociodemographic characteristics, was input as a control variable.

**Figure 1:**
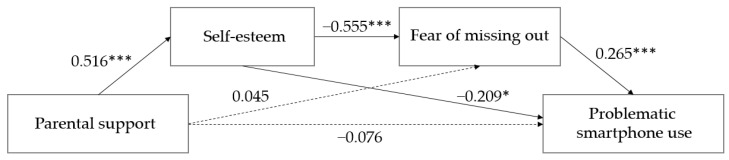
Serial mediation of self-esteem and fear of missing out in the relationship between parental support and problematic smartphone use with standard path coefficients. * *p* < 0.05; *** *p* < 0.001.

The results of verifying the significance of the model paths are shown in Table 3 and Figure 1. Parental support was found to be positively significant in self-esteem (B = 0.542, t = 9.023, *p* < 0.001), but it was not significant in FoMO (B = 0.061, t = 0.610, *p* = 0.543) and PSU (B = −0.053, t = −1.010, *p* = 0.314). Self-esteem was found to be significant in FoMO (B = −0.729, t = −7.250, *p* < 0.001) and PSU (B = −0.140, t = −2.349, *p* = 0.020), while FoMO was significant in PSU (B = 0.135, t = 3.624, *p* < 0.001).

Bootstrapping was performed to verify the significance of mediating effects on self-esteem and FoMO on the relationship between parental support and middle school students’ PSU. The results are shown in Table 4. The results show that the path of parental support reaching middle school students’ PSU going through self-esteem was significant (B = −0.076, CI (−0.142~−0.014)). Meanwhile, it was found that the path of parental support going through the FoMO to middle school students’ PSU was not significant (B = 0.008, CI (−0.018~0.042)). In addition, the path of parental support reaching PSU through self-esteem and FoMO was significant, as it did not include 0 in the 95% confidence interval of the mediating effects (B = −0.053, CI (−0.097~−0.018)).

## 4. Discussion

Based on problem behavior theory [12] and interpersonal acceptance–rejection theory [13], this study was designed to verify the potential mechanism that explains the relationship between parental support and adolescents’ PSU to set up a strategy for the prevention and management of adolescents’ PSU. The study examined what role self-esteem and FoMO play in the relationship mentioned above in detail.

To summarize the results, parental support was significantly indirectly associated with adolescents’ PSU through self-esteem and FoMO. It was in a positive relationship with adolescents’ self-esteem, which in turn influenced their PSU. Meanwhile, parental support did not have a direct effect on adolescents’ FoMO. These findings imply that parental factors play a significant role in adolescents’ PSU, also showing the importance of the mediative mechanism of self-esteem and FoMO.

In contrast to expectations, parental support did not have a direct effect on adolescents’ PSU (hypothesis 1). This is contrary to a preceding study that reported that parental support or the positive relationship between parents and children directly poses a negative effect on the adolescent’s PSU [48,49]. Such a result can be interpreted to have been driven by the developmental characteristics of the subjects. The subjects of this study were in early adolescence. Early adolescence is a period in which adolescents have more time spent alone with growing independence [50]; it is considered that their behaviors and lives are more likely to be indirectly influenced by parental support. For example, protective factors related to parents reinforce individual assets such as self-esteem, resilience, and positive thinking, affecting adolescents’ behaviors [51]. For the risk factors, there has been a number of studies on the relationship between parent-related factors (parental rejection, neglect, abuse, smartphone addiction, phubbing, etc.) and adolescents’ PSU [3]; however, as only a few studies have been conducted on parental factors as protective factors, there is a need for further research on their relationship.

The findings of this study show that self-esteem plays a mediating role in the relationship between parental support and PSU (hypothesis 2). In other words, parental support is positively related to self-esteem, which in turn affects adolescents’ PSU, supporting the results of preceding studies [23,27,52]. According to the IPAR theory [13], parental acceptance and support pose a positive influence on adolescents’ self-esteem, emotional stability, and viewpoints. On the contrary, adolescents who perceive rejection from their parents have their self-recognition process damaged, which leads them to negatively evaluate themselves [22]. The establishment of identity is one of the major developmental tasks in the adolescent period [53]. Adolescents who experience rejection, neglect, and low support from their parents go through a damaged self-defining process, which eventually leads to failure in the establishment of a positive identity, causing low self-esteem [54]. To recover the self-esteem damaged by relationships offline, adolescents with low self-esteem tend to focus on communicating with friends and partners on their smartphones, raising the risk of addiction [27,40]. Furthermore, low self-esteem can cause other negative cognitions and make emotional aspects vulnerable [40,52].

In contrast to expectations, parental support did not have a direct effect on adolescents’ FoMO; it was not consistent with hypothesis 3. Moreover, FoMO showed a positive relationship with adolescents’ PSU. In short, when there were two mediators—self-esteem and FoMO—in which parental support reduced adolescents’ FoMO by boosting their self-esteem rather than having a direct influence on it; as this, in turn, was found to be influencing adolescents’ PSU, the findings more strongly supported hypothesis 4 than 3. This implies that positive parental factors have an indirect effect on the FoMO. As adolescents have a developmental characteristic in which they regard establishing and maintaining bonds within their peer groups as important, they tend to overdepend on their smartphones if their FoMO on peer social relationships heightens [35,36,37]. Adolescents with increased FoMO may attempt to ease negative feelings by focusing on others’ experiences, building and maintaining relationships with them, and such behaviors can lead to PSU [55]. In addition, such adolescents tend to participate more heavily on social media without even noticing their psychological wellbeing; as this can create a vicious cycle, increasing the FoMO again, relevant management needs to take place [30,40].

As parental support was found to be having an indirect influence on adolescents’ PSU, mediating self-esteem and FoMO (hypothesis 4), the findings support the IPAR theory and the PBT that argue parental factors affect adolescent behaviors. As mentioned earlier, parents’ supportive and receptive nurturing attitudes help adolescents determine their relationship with parents and self-recognition, which can lead to their emotional wellbeing and social capacity [56,57]. While adolescents who perceive strong parental support build high self-esteem, those with weak parental support build low self-esteem, which leads to heightened social anxiety and a feeling of isolation [38,40]. Moreover, adolescents with a greater FoMO tend to overdepend on their smartphones to find psychological wellbeing [35]. As such, parental factors influence adolescents’ evaluation of themselves, identity problems, and emotional growth, which ultimately lead to behavioral aspects.

This study established a relationship between variables based on PBT [12] and IPAR theory [13]. The results were consistent with the IPAR theory; it was observed that adolescent self-esteem was positively influenced by parental support. Moreover, in line with the PBT, the perceived environmental system (parental support) influenced the adolescent personality system (self-esteem and FoMO) as well as the behavioral system (problematic smartphone use). In addition, as mentioned in previous studies, parents’ supportive and accepting nurturing behaviors contribute to adolescents’ emotional and psychological stability, which serves as a protective factor for adolescents’ problematic behaviors [11,20,21,22,23]. Greater efforts must, therefore, be made to improve the quality of parents’ childcare. Furthermore, in order to understand and improve the behavior of adolescents, not only personal factors but also environmental factors such as parents, teachers, and friends should be considered in an integrated manner.

The findings of this study suggest three strategies to reduce the risk of adolescents’ PSU. First, strong parental support was a significant protective factor for adolescents’ PSU. Hence, there is a need for a family support program for parents to create a supportive family environment along with assessment factors related to adolescents’ parents and families. In particular, education on how to nurture children to boost self-esteem needs to take place, and, hence, practitioners in medical facilities, schools, and communities should educate parents so that they can cultivate their capacity as parents through various methods, including lectures, counseling, and training. Second, the findings of this study show that high self-esteem is a protective factor against PSU. Therefore, school nurses should examine adolescents’ self-esteem first when intervening in the prevention of their PSU. There is also a need for the facilitation of programs to strengthen adolescents’ self-esteem. Third, as FoMO is a risk factor for PSU, it is necessary to ease such fear to prevent PSU. Therefore, this study suggests educators and school nurses be trained in counseling to reduce adolescent FoMO. In addition, as previous studies report that FoMO is affected by psychological factors such as depression, anxiety, and stress, there need to be various approaches taken for adolescents’ psychological wellbeing [29,31].

In this study, factors related to adolescents’ PSU were identified comprehensively as personal and environmental factors, and the mechanism between them was revealed. While existing studies put focus on the relationship between personal psychological factors and PSU, this study identified the mechanism applied between environmental factors (parental factors) and individuals’ cognitive and emotional factors, thereby expanding the knowledge about adolescents’ health and problematic behaviors. In addition, the findings provide theoretical grounds for the need to focus on parents’ nurturing attitudes, self-esteem, and FoMO when planning intervention programs related to adolescents’ smartphone use. Hence, it is expected that the results of this study can be used as primary evidence for the development of relevant programs.

Regardless of the significance of the study mentioned above, this study had a number of limitations. First, with the study being cross-sectional, only the relevance among the variables was identified; the cause-and-effect relationship among the variables cannot be explained. Therefore, the study suggests a longitudinal study be conducted to investigate the causal, mediating flow between parental and personal factors, as well as an ecological momentary assessment study to identify the relationship between each factor by measuring the temporal changes in parental factors, personal factors, and smartphone use. Second, as the data of the study were collected through self-report questionnaires, objectivity is limited, and there is a risk of desirability biases. Therefore, this study suggests further research to use a variety of methods (surveying, interviewing, and observing both parents and adolescents, etc.) to collect data with greater objectivity.

## 5. Conclusions

The findings of this study identified the roles of self-esteem and FoMO in the relationship between parental support and the PSU of adolescents. Self-esteem and FoMO, in particular, showed serial mediating effects. Such findings imply that the mechanism of adolescents’ PSU requires a complex consideration of factors, including parents’ nurturing attitudes and individuals’ cognitive and emotional factors. They also provide empirical evidence regarding the relationship between parents’ nurturing attitudes and PSU. Accordingly, appropriate strategies should be developed, targeting parents and adolescents to prevent and treat PSU among adolescents based on the results of this study. In detail, this study calls for the development of family support programs to create supportive family environments, as well as programs to strengthen adolescents’ self-esteem and counseling training for school nurses covering FoMO.

## Figures and Tables

**Table 1:** Problematic smartphone use by sociodemographic characteristics.

Characteristics	Categories	N	%	Problematic Smartphone Use	
				M ± SD	t or F (*p*)
Gender	Male	129	49.6	2.02 ± 0.52	−0.741 (0.459)
	Female	131	50.4	2.08 ± 0.55	
Age (year)	12	72	27.7	2.09 ± 0.59	0.416 (0.660)
	13	76	29.2	2.01 ± 0.54	
	≥14	112	43.1	2.08 ± 0.49	
Family structure	Living with both parents	218	83.8	2.07 ± 0.55	−0.711 (0.478)
	Living with one parent/ Not living with parents	42	16.2	2.00 ± 0.46	
Parents’ working status	Both parents are working	157	60.4	2.06 ± 0.55	0.096 (0.924)
	One of them is working/ None of them are working	103	39.6	2.05 ± 0.52	
Perceived economic status	High	69	26.5	1.97 ± 0.51	2.138 (0.121)
	Average	179	68.8	2.07 ± 0.54	
	Low	12	4.6	2.40 ± 0.34	
Perceived health status	Very healthy a	74	28.5	1.93 ± 0.53	5.128 (0.002) a < b
	Healthy b	139	53.5	2.19 ± 0.45	
	Unhealthy	41	15.8	2.18 ± 0.51	
	Very unhealthy	6	2.3	2.32 ± 0.38	

**Table 2:** Descriptive statistics and correlations between main variables.

**Variables**	**Min**	**Max**	**M**	**SD**	**1**	**2**	**3**
1. PS	1.13	5.00	3.93	0.76	-		
2. SE	1.30	5.00	3.59	0.80	0.550 ***	-	
3. FoMO	1.00	5.00	2.03	1.05	−0.259 ***	−0.519 ***	-
4. PSU	1.00	3.40	2.06	0.53	−0.269 ***	−0.413 ***	0.404 ***

PS, parental support; SE, self-esteem; FoMO, fear of missing out; PSU, problematic smartphone use; M, mean; SD, standard deviations; *** *p* < 0.001.

**Table 3:** Results of path analysis.

**Path**	**B**	**se**	***t***	***p***	**LLCI**	**ULCI**
PS → SE	0.542	0.060	9.023	<0.001	0.424	0.66
PS → FoMO	0.061	0.101	0.610	0.543	−0.137	0.26
SE → FoMO	−0.729	0.101	−7.250	<0.001	−0.927	−0.53
PS → PSU	−0.053	0.053	−1.010	0.314	−0.158	0.051
SE → PSU	−0.140	0.059	−2.349	0.020	−0.257	−0.022
FoMO → PSU	0.135	0.038	3.624	<0.001	0.061	0.209

PS, parental support; SE, self-esteem; FoMO, fear of missing out; PSU, problematic smartphone use.

**Table 4:** Bootstrapping analysis of the mediating model.

	**B**	**Boot SE**	**Boot LLCI**	**Boot ULCI**
Total indirect effect	−0.121	0.034	−0.189	−0.057
1. PS → SE → PSU	−0.076	0.033	−0.142	−0.014
2. PS → FoMO → PSU	0.008	0.015	−0.018	0.042
3. PS → SE → FoMO → PSU	−0.053	0.02	−0.097	−0.018

PS, parental support; SE, self-esteem; FoMO, fear of missing out; PSU, problematic smartphone use.

## Data Availability

Not applicable.
